# A Review on Microfluidics-Based Impedance Biosensors

**DOI:** 10.3390/bios13010083

**Published:** 2023-01-03

**Authors:** Yu-Shih Chen, Chun-Hao Huang, Ping-Ching Pai, Jungmok Seo, Kin Fong Lei

**Affiliations:** 1 Department of Biomedical Engineering, Chang Gung University, Taoyuan 33302, Taiwan; 2 Department of Radiation Oncology, Linkou Chang Gung Memorial Hospital, Taoyuan 33305, Taiwan; 3 Department of Electrical & Electronic Engineering, Yonsei University, Seoul 120-749, Republic of Korea

**Keywords:** microfluidic, impedance biosensor, electrical impedance flow cytometer, electrochemical impedance spectroscopy

## Abstract

Electrical impedance biosensors are powerful and continuously being developed for various biological sensing applications. In this line, the sensitivity of impedance biosensors embedded with microfluidic technologies, such as sheath flow focusing, dielectrophoretic focusing, and interdigitated electrode arrays, can still be greatly improved. In particular, reagent consumption reduction and analysis time-shortening features can highly increase the analytical capabilities of such biosensors. Moreover, the reliability and efficiency of analyses are benefited by microfluidics-enabled automation. Through the use of mature microfluidic technology, complicated biological processes can be shrunk and integrated into a single microfluidic system (e.g., lab-on-a-chip or micro-total analysis systems). By incorporating electrical impedance biosensors, hand-held and bench-top microfluidic systems can be easily developed and operated by personnel without professional training. Furthermore, the impedance spectrum provides broad information regarding cell size, membrane capacitance, cytoplasmic conductivity, and cytoplasmic permittivity without the need for fluorescent labeling, magnetic modifications, or other cellular treatments. In this review article, a comprehensive summary of microfluidics-based impedance biosensors is presented. The structure of this article is based on the different substrate material categorizations. Moreover, the development trend of microfluidics-based impedance biosensors is discussed, along with difficulties and challenges that may be encountered in the future.

## 1. Introduction

Biosensors are mainly used to measure or perceive signals from biological responses. Electrical biosensors can be generally classified into potential, current, and impedance sensors [1]. H. E. Ayliffe pioneered the measurement of single-cell impedance in a microchannel in 1999 [2]. Biological substances can be detected using a pair of microelectrodes with a gap of several μm, in a microchannel 10 μm in width. This narrow microchannel allowed for a more accurate impedance measurement of human polymorphonuclear leukocytes and teleost fish red blood cells. Subsequently, the electrical and equivalent circuit models of single cells were established [3,4].

As early as 1984, Giaever et al. designed a device on a Petri dish that could monitor impedance in order to investigate the density and cell migration of fibroblasts [5]. The impedance signal was obtained by gold electrodes 130 μm in width, which were deposited by a metal evaporator using a mask. Fibroblasts were attached to the gold electrodes, and their cellular response could be represented by the impedance measured across the electrodes [6]. The measured impedance values could differentiate normal fibroblasts from transformed cells. When fibronectin and gelatin were coated on the gold electrodes, the fibroblasts showed a better response. Based on the results of electrical signal monitoring, cell movement was observed. Later, Giaever published an article proposing that the phenomenon of cell movement is called micromotion [7], following which the authors put forward a mathematical model establishing the theory of cell movement analysis. Experimentally, small fluctuations in impedance electrical signals were measured as direct evidence of cell movement. Unlike cells observed by light microscopy, impedance biosensors are designed to continuously track the vertical movement of detected cells [8]. The range of cell motion can be as small as 1 nm. The impedance value and fluctuation of each type of cell differs and, so, may be used as a cell fingerprint. Regarding electrical impedance spectroscopy, Grossi et al. carried out a series of literature reviews [9]. The impedance spectrum is usually matched with an equivalent circuit model, and the impedance spectrum obtained by each test object can be expressed as the electrical fingerprint of the test object. Sun et al. used electrochemical impedance spectroscopy to explain the dielectric properties of individual polyelectrolyte microcapsules with different shell thicknesses [10]. The authors built a complete equivalent circuit model of a single solid spherical particle in suspension, and the resistance of the shell and the capacitance of the inner core were used to determine the permittivity and conductivity of individual capsule shells. The study indicated that the conductivity of the six- and nine-layer microcapsule shells could be estimated as 28 ± 6 and 3.3 ± 1.7 mS m^−1^, respectively.

On the other hand, the first microchannel-based flow cytometer was designed by Kamentsky et al. in 1965 [11], with a cell throughput of 500 cells per second. Initially, optical detection was used, using wavelengths of 253 nm and 410 nm. Later on, S. Gawad developed an impedance spectroscopy flow cytometer using Pt as an electrode, which could reduce the electrode impedance and allow the measurement frequency to be as low as 10 kHz [12]. By using a three-electrode design, the impedance value when the cells pass through can be easily measured. The peak impedance value is related to the cell size, and the electrode spacing and the time difference between two impedance signals could also be used to determine the velocity of the measured particle. Gawad et al. established a model to discriminate cell size, membrane capacitance, and cytoplasmic conductivity using a miniature cytometer [13]. Moreover, Cheung et al. obtained amplitude, opacity, and phase information using a microfluidic impedance cytometer, which can be used to distinguish different cells [14], where the measured amplitude can determine the cell size. Opacity was used to distinguish polystyrene beads from red blood cells (RBCs), while phase information was used to distinguish between RBCs and ghosts. RBCs and RBCs fixed in glutaraldehyde could also be distinguished by opacity. The definition of opacity was published in an article by R. A. Hoffman [15]; in particular, it is the ratio of the high-frequency impedance to low-frequency impedance of a particle. Some scholars have studied the development of fluidic impedance cytometers for micro-single cells [16].

Impedance biosensors integrated with microfluidic technology can be categorized into two major technologies: electrical impedance flow cytometry and electrochemical impedance spectroscopy. In the design of a microchannel, an electrical impedance flow cytometer can measure dynamic objects using impedance technology. On the other hand, electrochemical impedance spectroscopy is usually combined with microcavity structures. Static objects can be measured using impedance measurement techniques. In this review article, microfluidics-based impedance biosensors are comprehensively summarized and classified according to the different substrate materials. The development trend, difficulties, and challenges associated with microfluidics-based impedance biosensors, which are expected to be encountered in the future, are also discussed.

## 2. Silicon-Based Impedance Biosensors

Silicon-based impedance biosensors are only possible with the fabrication of the electrodes by micromachining. Therefore, the development of silicon impedance biosensors began earlier; however, a number of articles have also been published recently, due to relevant special processes.

**Figure 1:**
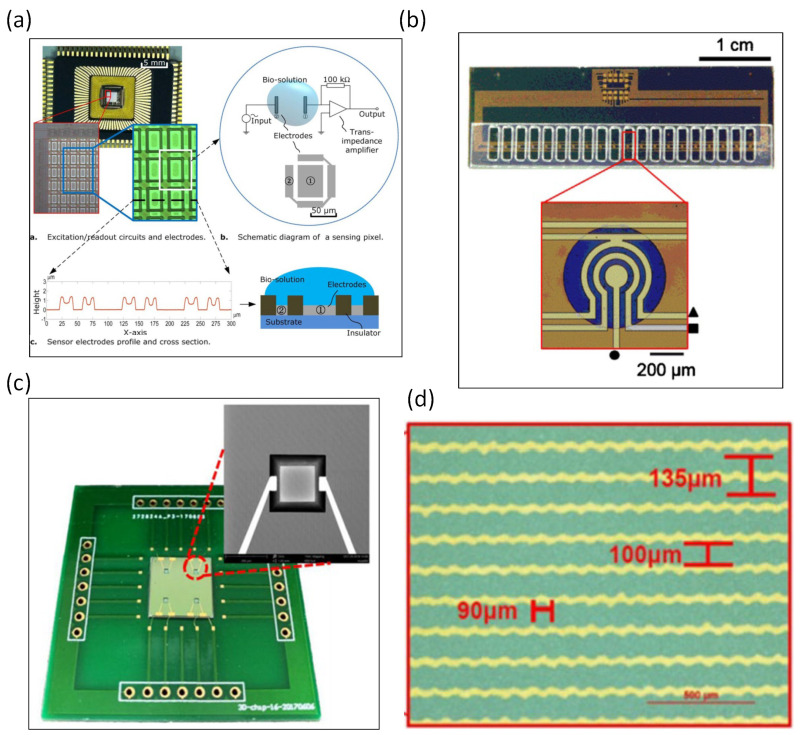
The silicon-based impedance biosensors. (**a**) A low-cost 0.35 mm CMOS technology by TSMC (Taiwan) was used to fabricate the micro-array chip that sensed DNA characterization. Reproduced with permission from [27]. Copyright Scientific Reports 2013. (**b**) Integrated surface plasmon resonance and electrochemical impedance spectroscopy in a microfluidic chip [30]. Reproduced with permission from [30]. Copyright Sensors and Actuators B: Chemical 2017. (**c**) Silicon substrates were microfabricated to create microgrooves. Reproduced with permission from [32]. Copyright Microsystems and Nanoengineering 2020. (**d**) A biosensor combining impedance and photoelectrochemical was designed. Reproduced with permission from [33]. Copyright Biosensors 2022.

An impedance biosensor with a nanometer-wide interdigitated electrode array was developed [17], with electrode widths and spacings of 250–500 nm microfabricated using deep UV lithography. The same article also verified the binding of biomolecular structures to nanoscale electrode surfaces. The impedance signal results indicate that the immobilization of glucose oxidase on the electrode can be monitored. A silicon-based microfabricated biochip was designed to measure the electrical impedance spectrum [18], which could measure the conductance change in a 30 nl volume of bacterial suspension and showed the viability of the bacteria. The same research demonstrated electrical impedance values for the live micro-organism *Listeria innocuous*. By-products after bacterial metabolism have also been shown to change the electrical impedance value; for example, Radke et al. developed an impedance biosensor for bacterial detection using immobilized antibodies. The interdigitated electrode arrays were designed on silicon-based biosensors [19]. *Escherichia coli*-specific antibodies were immobilized on the electrodes, and impedance changes due to bacteria immobilized on the interdigitated gold electrodes were observed. Impedance signals at low frequencies showed that bacteria bound to the sensor electrode surface within 5 min. The rate of binding was the most pronounced in the first 20 min but slowed down significantly after 35 min. At high frequencies, impedance does not change over time. Test concentrations can be as low as 10 CFU/mL of bacterial suspension. The same research team used an impedance biosensor with immobilized antibodies and interdigitated electrode arrays to detect pathogenic *Escherichia coli* O157:H7 and Salmonella infants [20]. It mainly detects bacteria in food or water. P-type (100) silicon wafers were used in that article. Coplanar impedance sensors were designed on glass and fabricated by photolithography [21]. They verified that the spacing of the set of coplanar electrodes is a more important parameter than the electrode area. As the spacing of the electrode design increases, the impedance value increases accordingly. An impedance sensor was used to monitor drug-affected spheroids in a microcavity [22]. Silicon wafers were microfabricated into microcavities through wet anisotropic etching. The impedance sensor was designed with 15 microcavities, and 4 electrodes in each cavity were used to sense the impedance of the spheroids. OLN93 cell spheroids were most loosely organized and peaked at around 180 kHz, while Bro cell spheroids had a more compact structure and showed a peak at 100 kHz. They also observed the impedance of the spheroids 8 h after drug administration. Impedance increased with forskolin, camptothecin, and staurosporine and decreased with doxorubicin and tamoxifen. Single-cell impedance and optical sensing were integrated into a single chip for real-time viability assessment [23]. Single-cell capture microwell chips were obtained by etching silicon wafers with KOH. The induction chip is composed of two cavities (upper and lower). The adhesion changes of RAW264.7 macrophages can be assessed through the impedance value of the sensing chip. In an interdigitated electrode array, the gap between the electrodes is an important factor to improve the sensitivity of the biosensor [24]. Three-dimensional interdigitated electrode arrays were fabricated to sense the impedance signals of proteins. C-reactive protein-specific antibodies were immobilized on the electrode surface. The results showed that electrochemical impedance spectroscopy can be used to monitor the concentration of C-reactive protein in human serum. An impedance biosensor that can sense the concentration of picesterol was developed [25]. The electrodes on the silicon wafer were designed with interdigitated electrodes, where the distance between two electrodes was 15 μm. Cortisol-specific monoclonal antibodies were immobilized on the surface of the microelectrodes. Cortisol binding to antibodies can be signaled by changing the impedance values. The experimental results showed that the impedance biosensor can accurately detect cortisol in the range of 1 pM to 10 nM in saliva. An electrical impedance biosensor was constructed through a CMOS-process, using high-density sensing electrodes for the detection of breast tumor cells (MCF-7) [26]. A total of 96 × 96 gold microelectrodes were designed with a sensing area of 3.5 mm × 3.5 mm. The impedance signal is read out by an integrated circuit fabricated with 0.18 μm CMOS technology. The results showed that the increase in impedance was associated with cell binding to the electrode surface. Ma et al. designed an impedance biosensor that can detect suspended DNA, as shown in Figure 1a [27], where the sensing electrodes are fabricated using 0.35 μm CMOS technology. They proposed that the impedance of a solution is highly dependent on the concentration. Moreover, the impedance value of the sample solution is also highly correlated with the length of the DNA fragment. In other words, the biological samples obtained after PCR can be tested using the biosensors designed in that article. Lisandro Cunci developed electrochemical impedance biosensors that can detect telomerase in cancer cells [28]. The flexible heater and temperature sensor were designed together in the biosensor. Single-stranded DNA probes were immobilized on the surface of the interdigitated gold electrode array. Jurkat cells were tested for telomerase and showed a 14-fold increase in electrical resistance. The sensitivity of electrochemical impedance spectroscopy biosensors was enhanced using electric field focusing of magnetic beads in microwells [29], where the antibody is immobilized on the surface of the magnetic beads. Microwells are fabricated on silicon wafers using high aspect ratio SU-8 microstructures. In the same article, prostate-specific antigen in a PBS buffer and human plasma were used to validate the argument that focused magnetic beads can improve the sensitivity. The experimental results showed that prostate-specific antigens at low concentrations (i.e., tens or hundreds of fg/mL) could be detected. Pursey et al. integrated surface plasmon resonance and electrochemical impedance spectroscopy into a microfluidic chip, targeting bladder cancer-associated DNA sequences, as shown in Figure 1b [30]. Gold electrodes are microfabricated on the silicon layer, and 20 sensors on the same wafer simultaneously detect three different DNA markers for bladder cancer. Signals were measured within a short period of 20 min. Impedance biosensors that can monitor bacteria have also been developed. In one study, 216 three-dimensional interdigitated electrodes of 3 μm width and with 3 μm gap were microfabricated on a silicon wafer [31]. To improve the sensitivity, the three-dimensional electrodes were separated by an insulating layer. In addition to sensing the impedance signals of bacteria, the three-dimensional electrode can also concentrate bacteria. An impedance sensor fabricated in microgrooves was developed, where silicon substrates were microfabricated to create the microgrooves, as shown in Figure 1c [32]. Two gold electrodes were microfabricated in the microgrooves, which can be used to sense the impedance value of three-dimensional cancer cells, and changes in impedance can reflect the proliferation and apoptosis of three-dimensional cancer cells. Impedance sensors can also identify cancer cells that are affected by drugs. A biosensor combining impedance and photoelectrochemical analysis of cancer cells was designed, as shown in Figure 1d [33], where monocrystalline silicon was used as a substrate for the biosensor. The serrated interdigitated electrodes not only can focus cells but also sense impedance signals. They identified four different types of cancer cells: esophageal cancer (CE81T cell), esophageal cancer (OE21 cell), lung adenocarcinoma (A549 cell), and bladder cancer (TSGH-8301 cell).

The development of silicon chips for microfluidic impedance sensors depends on the development of the microelectromechanical system. Many silicon-based microfabrication processes and metal microelectrode processes are derived from microelectromechanical system microfabrication. Therefore, this is discussed in the first part of this article. Due to the special process requirements, several articles have focused on silicon-based microfluidic impedance sensors. In recent years, the COVID-19 virus has spread throughout the world, and this multi-mutated virus needs to be sensed through the use of biosensors. The MEMS process developed by Taiwan’s Taiwan Semiconductor Manufacturing Company may provide the direction for such a technology to be commercialized.

## 3. Printed Circuit Board (PCB)-Based Impedance Biosensors

Printed circuit boards (PCBs) are flat plates that were originally used to make circuits, which are very commercialized. Their electrode width and electrode spacing are not very small; therefore, the sensitivity limit of the sensor is not low either.

**Figure 2:**
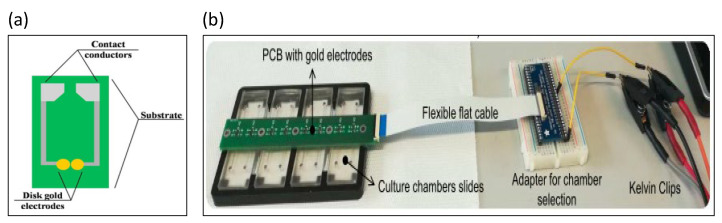
The PCB-based impedance biosensors. (**a**) Gold electrodes were printed on commercial printed circuit boards. Reproduced with permission from [35]. Copyright Scientific Reports 2013. (**b**) An electrical impedance sensor was used to detect the degree of fibrosis in liver tissue. Reproduced with permission from [37]. Copyright Biosensors 2022.

Electrochemical impedance spectroscopy biosensors consist of PCBs with gold-coated electrodes, which are mainly used to detect plant pathogens [34], where the antibody is first bound to the surface of the electrochemical sensor. For papaya ring spot virus, the biosensor was shown to be capable of detecting papaya ring spot virus coat protein with high sensitivity. A chip using a microfluidic impedance sensing system to detect the transgenic protein Cry1Ab was designed, as shown in Figure 2a [35]. Gold electrodes were printed on commercial printed circuit boards, with the spacing between two printed electrodes being 250 μm. The impedance signal at the optimal test frequency (358.3 Hz) presented a good linear relationship with the concentration of the transgenic protein Cry1Ab in the range of 0–0.2 nM. Clinically, the degree of red blood cell agglutination is divided into five grades by visual inspection, which is routinely conducted in hospitals [36]. An electrical impedance blood-sensing chip was designed by Chang et al. to distinguish the degree of blood agglutination. The interdigitated electrode array was designed on a PCB. ZnO nanowires were synthesized on the surface of the electrode array in order to improve the sensitivity of impedance measurement. An electrical impedance sensor was used to detect the degree of fibrosis in liver tissue, as shown in Figure 2b [37], where liver tissue was detected by a pair of gold electrodes on the PCB. The experimental results indicate that the maximum resistance difference between healthy and fibrotic tissue was about 2 kΩ at day 8.

The development of PCB chips is limited by the electrode line width in traditional PCB processes. Microelectrodes that are not tiny enough will lead to an inability to improve the sensitivity. Therefore, PCB chips are more suitable for the production or commercial design where the induction is fast and the sensitivity requirements are not high.

## 4. Polymer-Based Impedance Biosensors

In addition to glass, the most commonly used substrates in the field of microfluidics are polymer chips. Different from the stretchable chips mentioned in later chapters, the polymer chips mentioned in this chapter are formed of hard and inflexible materials.

**Figure 3:**
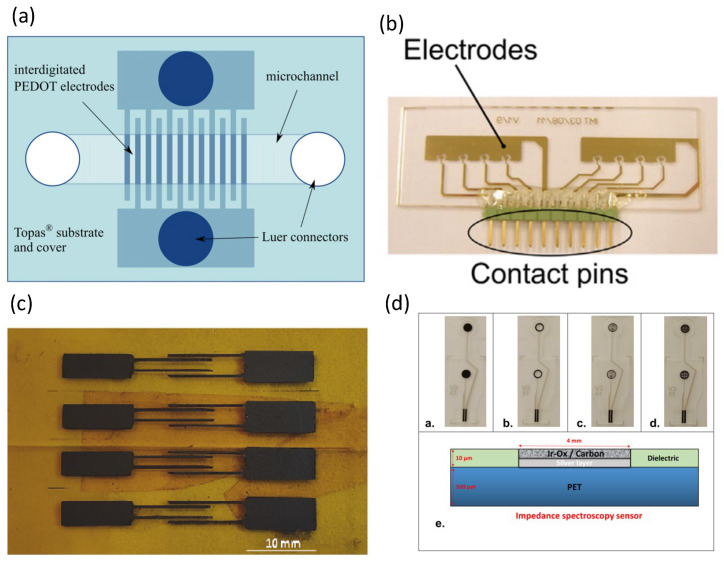
The polymer-based impedance biosensors. (**a**) All-polymer electrochemical microfluidic biosensors are used. Reproduced with permission from [40]. Copyright Biosensors and Bioelectronics 2013. (**b**) Two microfluidic chambers were designed on a cyclic olefin copolymer substrate. Reproduced with permission from [41]. Copyright Biosensors and Bioelectronics 2013. (**c**) Kapton Polyimide (PI) was used as a substrate. Reproduced with permission from [47]. Copyright Biosensors and Bioelectronics 2022. (**d**) Microelectrodes were microfabricated on polyethylene terephthalate (PET) sheets. Reproduced with permission from [48]. Copyright Biosensors 2022.

A study considering electrochemical impedance spectroscopy on polymer substrates was developed [38], in which the authors designed a nanoscale interdigitated electric shock array, in which the electrode width was only 200 nm and the electrode spacing was 500 nm. Gold nanometer interdigitated electrode arrays were patterned on cyclic olefin copolymer substrates. Experiments have demonstrated selective iDEP capture and impedance detection on polystyrene microspheres and *Bacillus subtilis* spores [39]. The authors used oxides to passivate the sensing electrode of the sensor in order to avoid the metal and electrolyte having adverse effects on the electrode surface. Cyclic olefin copolymer was used as the substrate of the sensor. Studies have used all-polymer electrochemical microfluidic biosensors for electrochemical impedance spectroscopy, as shown in Figure 3a [40], where polymer materials from Topas Corporation were used as substrates and conductive polymer bilayers were used as electrode materials. Electrochemical impedance spectroscopy was able to detect ampicillin in a concentration range from 100 pM to 1 μM and kanamycin A from 10 nM to 1 mM. Figure 3b shows how Pires et al. combined an impedance sensor and a current sensor to detect biofilms in water [41]. Two microfluidic chambers were designed on a cyclic olefin copolymer substrate with four impedance sensors and three current sensors in each cavity. A conductive polymer (PEDOT:TsO) was fabricated as an interdigitated electrode array for impedance biosensors [42], where the cyclic olefin copolymer produced by TOPAS was used as the base material for the conductive polymer electrodes. A microfluidic impedance sensor was used to detect the food additive clenbuterol hydrochloride [43], where the electrodes were patterned on poly (ethylene terephthalate) films. Polyaniline@graphene oxide nanocomposites were used to functionalize the sensing electrodes, and the microfluidic impedance sensors could detect down to 0.12 ppb. Sharif et al. integrated a microfluidic system and a magnetic separation procedure to develop a novel impedance sensor for the detection of various foodborne pathogens [44]. Their results showed that the impedance sensor was effective for the detection of various foodborne pathogens, including *Escherichia coli* (*E. coli* O157:H7), *Vibrio parahaemolyticus* (*V. parahaemolyticus*), *Staphylococcus aureus* (*S. aureus*), and *Listeria monocytogenes* (*L. monocytogenes*). Polymethyl methacrylate (PMMA) was used as the substrate. D-dimer is a biomarker in the blood that can be used to diagnose deep vein thrombosis and pulmonary embolism [45]. Lakey et al. designed a polymer microfluidic impedance sensor for the detection of D-dimer, where interdigitated electrode arrays were patterned on polyethylene naphthalate (PEN) substrates. Ma et al. developed an electrochemical impedance spectroscopy approach for the detection of endotoxins [46]. The electrodes were screen-printed on a polyethylene terephthalate (PET) substrate, which contained carbon for the working and auxiliary electrodes and Ag/AgCl for the reference electrode. The sensitivity of the impedance biosensors could be as low as 500 pg mL^−1^, and the total measurement time was only half an hour. Niaraki et al. used graphene microelectrodes to monitor neuronal growth and detachment after death, as shown in Figure 3c [47]. Kapton Polyimide (PI) was used as a substrate for patterned graphene electrodes. The microelectrodes fabricated by this research team feature a wrinkled surface morphology, which allows for a fast response time to be achieved. Chmayssem et al. integrated a cell culture chamber with electrochemical impedance spectroscopy, as shown in Figure 3d [48]. The researchers used a screen-printing technique to fabricate the microelectrodes on polyethylene terephthalate (PET) sheets. The electrode material was selected, and Ag/AgCl was used as the low interface impedance electrode on the PET sheet. On the top layer of electrodes was carbon-biocompatible ink rich in IrOx particles. Hantschke et al. integrated electrophoretic and electrical impedance sensors for point-of-care (POC) diagnostics [49], where the electrodes and microchannels were fabricated on polymethylmethacrylate (PMMA) substrates.

The key to the use of polymer chips in microfluidic impedance biosensors is the combination of microelectrodes and polymer substrates. The sensitivity of the biosensors were determined by the size of the microelectrodes. Polymer chips are used similarly to glass chips, with both being hard and inelastic materials. On the other hand, polymer materials are suitable for mass production. Therefore, polymer chips can be one of the options for commercialization.

## 5. Glass-Based Impedance Biosensors

Microfluidic chips with glass as the substrate are very common. There have been some articles on materials that can replace glass, such as ITO glass, Pyrex glass, and SiO_2_ glass. Therefore, the classification in this chapter is based on what the biosensors on glass can monitor. In this line, the subsections are classified regarding the detection of bacteria, blood, cells, DNA, proteins, toxins, and viruses.

### 5.1. Detection of Bacteria

**Figure 4:**
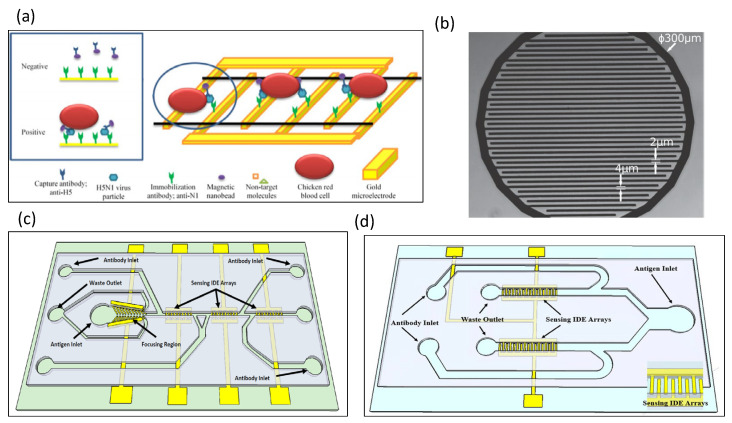
Glass-based impedance biosensors for detecting bacteria. (**a**) A polyclonal antibody was immobilized on the surface of a microelectrode and bound to avian influenza virus H5N1 to generate an impedance signal. Reproduced with permission from [55]. Copyright Biosensors and Bioelectronics 2012. (**b**) Pyrex wafers were used as substrates for impedance sensors. Reproduced with permission from [57]. Copyright Biosensors and Bioelectronics 2015. (**c**) An impedance-based biosensors were used to simultaneously detect Salmonella Serotypes B, D, and E. Reproduced with permission from [58]. Copyright Scientific Reports 2018. (**d**) There were two sensing areas in the chip, and the sensing electrodes were composed of interdigitated electrodes. Reproduced with permission from [60]. Copyright PLoS ONE 2019.

Ruan et al. used electrochemical impedance spectroscopy to detect *E. coli* O157:H7 [50]. An anti-*E. coli* O157:H7 antibody was immobilized on the surface of an indium tin oxide (ITO) electrode. The binding of the antibody to the antigen changed the impedance signal. The limit of the sensor to detect bacteria was as low as 6 × 10^3^ cells/mL. Yang et al. used an impedance sensor to detect Salmonella typhimurium and observed impedance changes during bacterial growth [51]. The material of the interdigitated electrode array was ITO. Four frequencies (10 Hz, 100 Hz, 1 kHz, and 10 kHz) were used to record impedance growth curves in the experiment. The impedance changes only when the bacterial count reaches 10^5^–10^6^ CFU/mL. Experimental data indicated that the greatest impedance change was observed at 10 Hz. [Fe(CN)6]^3−/4−^ was used as a redox probe [52], which increases the electron transfer resistance on the antibody-immobilized microelectrode surface. Impedance immunosensors for the detection of Listeria were developed [53], for which TiO_2_ nanowires were immobilized on gold electrodes, and monoclonal antibodies were immobilized on the nanowires. Listeria was then specifically captured using the antibodies. The impedance immunosensors sense impedance changes induced by the nanowire–antibody–bacteria system. Tan et al. designed a microfluidic impedance immunosensor for the detection of *Escherichia coli* and *Staphylococcus aureus* [54]; in particular, specific antibodies were immobilized on alumina nanoporous membranes, and bacteria were captured on the nanoporous membranes by antibodies. In a 2 h rapid assay, the sensitivity was as high as 10^2^ CFU/mL. J. Lum designed an impedance biosensor, mainly for avian influenza virus subtype H5N1, as shown in Figure 4a [55]. Immunomagnetic nanoparticles and interdigitated microelectrodes were designed on a microfluidic chip. The polyclonal antibody was immobilized on the surface of the microelectrode, which binds to the avian influenza virus H5N1 to generate an impedance signal. Chicken red blood cells were used as biomarkers attached to the interdigital microelectrodes. The results showed that the impedance signal was increased by more than 100%. An impedance biosensor with focusing and sensing electrodes was designed to detect *E. coli* O157:H7 [56]. The sensitivity lower limit of detection for impedance sensor measurements was 3 × 10^2^ CFU/mL. By focusing on p-DEP, the measurement sensitivity can be increased by 2.9 to 4.5 times. Couniot et al. integrated a CMOS process to design an impedance sensor for bacterial detection in urine, as shown in Figure 4b [57], where the detection of impedance spectroscopy was mainly directed at *Staphylococcus epidermidis*. Pyrex wafers were used as substrates for the impedance sensors. Liu et al. developed impedance-based biosensors that can be used to simultaneously detect Salmonella Serotypes B, D, and E by relying on three sensing IDE arrays, as shown in Figure 4c [58]. The sensor was designed with focusing electrodes to generate dielectrophoretic force, where the focusing force can increase the sensitivity by 4–4.5 times, and the detection limit is as low as 8 cells/mL. Other results indicated that the sensor could also distinguish between dead and live cells. Dastider et al. designed a microfluidic impedance sensor that can sense low concentrations of *E. coli* O157:H7 [59]. Upstream of the microchannel, the authors designed interdigitated focusing electrodes, in which the electrodes were arranged in a 45-degree-inclined manner. Positive DEP forces were used to focus cells in the center of the microchannel. Then, downstream of the microchannel, three sets of interdigital electrode arrays (IDEAs) were designed to sense impedance. An *E. coli* antibody was functionalized on the sensing electrode, which captured *E. coli* and resulted in a change in impedance. Experiments have shown that the microfluidic impedance sensor can detect coliform bacteria at a concentration of 39 CFU/mL. A microfluidic impedance sensor was used to detect Salmonella Serotypes B and D in food, as shown in Figure 4d [60]. There are two sensing areas in the chip, and the sensing electrodes are composed of interdigitated electrodes. The shocks were coated with antibodies against Salmonella. The experimental results demonstrate that the impedance sensor can detect Salmonella as low as 300 cells/mL.

Many of the keys aspects in the design of impedance biosensors used to sense bacteria are related to improving the sensitivity. As the combination of antigen and antibodies can lead to changes in the impedance signal, many studies on antibodies immobilized on electrodes have been carried out.

### 5.2. Detection of Blood Samples

**Figure 5:**
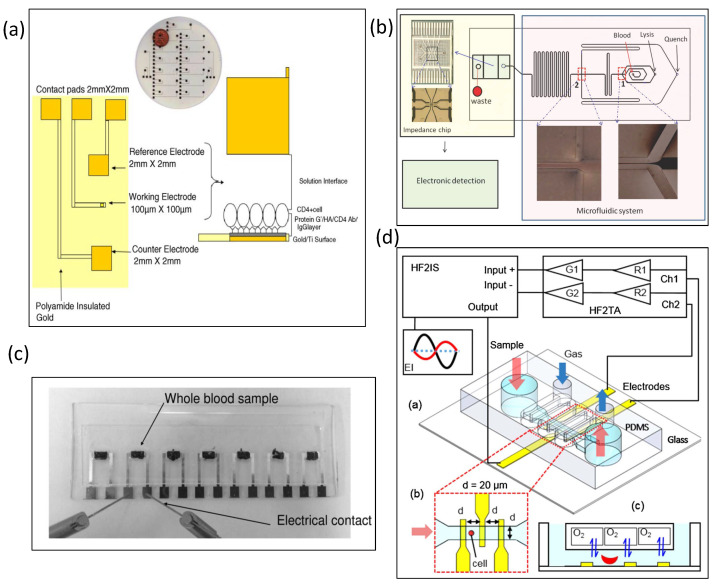
Glass-based impedance biosensors for detecting blood. (**a**) Three microelectrodes were used to sense the impedance of human CD4(+) cells in blood. Reproduced with permission from [61]. Copyright Biosensors and Bioelectronics 2005. (**b**) A microfluidic impedance cytometer was for red blood cell lysis. Reproduced with permission from [64]. Copyright Analytical Chemistry 2011. (**c**) A microfluidic chip was designed to detect electrical impedance for monitoring the blood coagulation process. Reproduced with permission from [65]. Copyright PLoS ONE 2013. (**d**) An electrical impedance microflow cytometer with controlled oxygen flow for the analysis of sickle blood cells. Reproduced with permission from [69]. Copyright Sensors and Actuators B: Chemical 2018.

The manufacturing of electrical impedance measurement systems became possible with microfabrication technology. For example, wet etching was used to obtain microchannels on a coverslip [2], and gold electrodes were plated on both sides of the microchannel. The authors scanned the electric impedance spectroscopy of ionic salt solutions, air, and deionized (DI) water in the frequency range from 100 Hz to 2 MHz. Their experimental results showed the efficacy of electrical impedance spectroscopy for human polymorphonuclear leukocytes and teleost fish red blood cells. Mishra et al. used three microelectrodes on a chip to sense the impedance of human CD4(+) cells in blood, as shown in Figure 5a [61]. The reference electrode, working electrode, and counter electrode were microfabricated on glass wafers. As the protein adsorbed onto the microelectrode surface, the detected impedance value increased even more. The impedance also increased with the number of captured cells. Kuttel et al. used impedance spectroscopy to detect red blood cells infected with *Babesia bovis* [62]. The change in impedance was mainly due to the presence of the parasite in the cell changing the impedance value of the original red blood cells, white blood cells, or platelets. Therefore, infected cells could be easily and quickly distinguished from healthy cells. In areas without good infrastructure or in very remote areas, the use of impedance spectroscopy to detect parasites in whole blood samples can greatly reduce the time of diagnosis for medical personnel. Holmes et al. developed a high-speed microfluidic single-cell impedance cytometer using dual frequency for whole blood analysis [63], which is mainly used for the impedance measurement and identification of T lymphocytes, monocytes, and neutrophils. The experiments showed that, at low frequencies, T lymphocytes and neutrophils can be distinguished. The cells were conjugated with fluorescently labeled antibodies, allowing the system to analyze fluorescence and impedance simultaneously. Han et al. developed a microfluidic chip that integrates red blood cell lysis with a microfluidic impedance cytometer, as shown in Figure 5b [64]. Their laboratory developed a buffer that not only lyses red blood cells but also increases the identification of monocytes and neutrophils. The method for multi-step cell lysis described in this paper is of great help for the microfluidic system, in terms of whole blood analysis. Lei et al. designed an electrical impedance to monitor the blood coagulation process in a microfluidic chip, which can obtain impedance results consistent with clinical reports at different temperatures and blood cell counts, as shown in Figure 5c [65]. This device provides a new analytical method for the sensitive and real-time monitoring of coagulation in whole blood samples. Song et al. developed a microfluidic impedance flow cytometer to identify undifferentiated and differentiated mouse embryonic stem cells [66], where two micropores and three electrodes were designed in the chip. The experimental results indicated that undifferentiated stem cells and polystyrene spheres could be distinguished at any frequency, while undifferentiated and differentiated stem cells require higher frequency and opacity to be distinguished. Du et al. designed an electrical impedance flow cytometer targeting red blood cells infected with *Plasmodium falciparum* [67]. The physiological and electrical properties of erythrocytes were altered 48 h after *P. falciparum* infection. In addition to the cytometer, the authors incorporated new offset parameters to make it easier to distinguish infected erythrocytes from uninfected erythrocytes. Spencer et al. used a microfluidic impedance cytometer for the detection of a representative circulating tumor cell (the MCF7 tumor cell line) [68]. The red blood cells were removed by lysis, and the buffer did not affect the dielectric properties of the MCF7 cells. Through impedance analysis, MCF7 cells were shown to have a larger size and membrane capacitance. The experimental results indicated that 100 MCF7 cells could be detected in 1 mL of whole blood. The average recovery rate was as high as 92%. Liu et al. developed an electrical impedance microflow cytometer that can control oxygen flow for the analysis of sickle blood cells, as shown in Figure 5d [69]. The two-layer microfluidic channel was separated by a 150 μm thick PDMS film. The upper layer is a serpentine gas channel that controls oxygen, while the lower layer is a microchannel through which sickle cells and red blood cells flow. Ti/Au electrodes were designed to measure the impedance of sickle cells in the lower microchannel. Under normoxic conditions, the authors distinguished between normal and sickle cells using impedance signals measured at intermediate frequencies. The same research demonstrated that impedance signals can be obtained without the need for hemolysis. Their experimental results also proved that the impedance signal of the microflow cytometer can be used as an indicator of red blood cell disease and sickle cell disease.

### 5.3. Static Cell Analyzed by Electrical Impedance Spectroscopy

**Figure 6:**
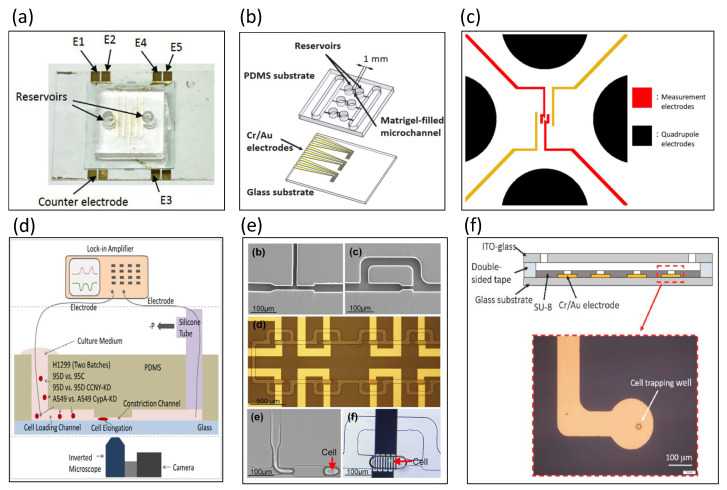
Static cell analyzed with electrical impedance spectroscopy. (**a**) A microchannel was designed filled with Matrigel to quantify cell migration velocity. Reproduced with permission from [72]. Copyright Analytica Chimica Acta 2020. (**b**) This chip was designed to induce angiogenesis to extend into the microchannel. Reproduced with permission from [73]. Copyright Sensors and Actuators B: Chemical 2022. (**c**) The relationship between the electrochemical impedance spectroscopy and withstand voltage was established with four different cells (HeLa, A549, MCF-7, and MDA-MB-231). Reproduced with permission from [74]. Copyright Sensors and Actuators B: Chemical 2012. (**d**) Because of the constriction of the microchannel, tumor cells, thus, are elongated for sensing impedance. Reproduced with permission from [75]. Copyright Biosensors and Bioelectronics 2014. (**e**) A microfluidic impedance sensing chip with droplets and microelectrode arrays was used to monitor the osteogenic differentiation of bone marrow mesenchymal stem cells. Reproduced with permission from [76]. Copyright Biosensors and Bioelectronics 2019. (**f**) Single stem cells were captured in a 20 μm chamber by dielectrophoresis. Reproduced with permission from [77]. Copyright Talanta 2021.

A microfluidic impedance sensor was designed to measure the cell migration of cancer cells in a three-dimensional extracellular matrix [70]. A total of 16 sets of sensing electrode arrays and cell grabbing arrays were designed in the microchannel. Under continuous monitoring, the migration of MDA-MB-231 cells allowed for a rapid change in impedance amplitude (of about 10 Ω/s). Liu et al. designed a microfluidic chip with embedded measurement electrodes to monitor the cell migration process using impedance measurement technology [71]. Cells were measured and recorded in the microfluidic channel as they pass through multiple parallel electrodes. This method enables the accurate and objective recording of cell migration activity and the calculation of migration rates among different stimulating drugs. Huang et al. designed a microchannel filled with Matrigel to quantify cell migration velocity as an assay tool, as shown in Figure 6a [72]. The successful measurement of cells suspended in a 3D environment and the induction of cell migration by stimulatory factors were used to record the migration speed of cells. The measurement sensitivity was better than that of a traditional trans-well assay.

Lei et al. developed a perfusion three-dimensional (3D) cell culture microfluidic chip combined with real-time and non-invasive impedance monitoring [73]. This device can simulate complex 3D biological microenvironments to culture cells and monitor the impedance changes under different concentrations of drug stimulation through impedance measurements. The impedance results are analyzed to determine the cell proliferation and chemosensitivity of 3D cell cultures. Lei et al. designed an impedance measurement device for cell colonies cultured on hydrogels [74,75,76]. Huang et al. constructed a 3D biological barrier using Matrigel and induced angiogenesis to extend into the microchannel, as shown in Figure 6b [77]. The angiogenesis process could be monitored by label-free impedance, using electrodes at the bottom of the microchannel. The device can also successfully quantify the time and distance of angiogenesis, thereby providing a reliable and quantitative method for the assay of angiogenesis.

Bieberich et al. developed electrical cell impedance spectroscopy to monitor the impedance response of PC12 and embryonic stem cells forming synapses [78]. Jang et al. published a study combining a cell capture method with microfabricated impedance spectroscopy [79], in which three micropillars were designed in the center of the microchannel to capture single HeLa cells. Cho et al. detailed the integrated microfluidic capture of single-cell technology with electrical impedance spectroscopy [80]. Hildebrandt et al. developed electrochemical impedance spectroscopy to distinguish the osteogenic differentiation of human mesenchymal stem cells [81]. In the application of cellular impedance to infectious parasites, Houssin et al. designed an electrochemical impedance spectroscopy approach to detect the presence of oocysts [82]. Dalmay et al. developed impedance spectroscopy to distinguish cancer stem cells and U87 glial cells (differentiated cells) [83]. The impedance spectrum designed by Bagnaninchi et al. can instantly monitor adipose stem cell (ADSC) differentiation [84]. Hong et al. established the relationship between electrochemical impedance spectroscopy and the withstand voltage of four different cells (HeLa, A549, MCF-7, and MDA-MB-231), as shown in Figure 6c [85]. Under a strong electric field, the cytoplasmic resistance decreases due to the opening of ion channels. The experimental results showed that different cells have not only different impedance spectra but also different withstand voltages. Chen et al. designed a microfluidic chip for capturing single cells and measuring impedance values [86]. Zhao et al. proposed to convert the measured impedance values into membrane capacitance (C-specific membrane) and cytoplasmic conductivity (σ cytoplasm), as shown in Figure 6d [87]. Due to the constriction of the microchannel, tumor cells become elongated. The experimental results demonstrated that tumor cells can be distinguished using two parameters: C-specific membrane and sigma cytoplasm. Ruan et al. integrated dielectrophoretic force and impedance sensors to detect lung circulating tumor cells [88]. Fan et al. designed a microfluidic impedance sensing chip with droplets and microelectrode arrays to monitor the osteogenic differentiation of bone marrow mesenchymal stem cells, as shown in Figure 6e [89]; the authors also proposed a model of cellular droplets. Lei et al. captured single stem cells in a 20 μm chamber by dielectrophoresis, as shown in Figure 6f [90]; the cells were measured in the impedance spectrum range of 2–20 kHz.

### 5.4. Dynamic Cell Analyzed by Microfluidic Impedance Cytometry

The development of microfluidic impedance flow cytometry is important for cell analysis. In this subsection, microfluidic impedance flow cytometry is divided into two parts for discussion: one is the principle of microfluidic impedance flow cytometry, and the other includes the sensing applications of microfluidic impedance flow cytometry.

Sun et al. designed two microfluidic impedance cytometers with parallel facing electrodes and coplanar electrodes [91]. For impedance measurements, parallel facing electrode designs are more sensitive than coplanar electrode designs. Holmes et al. used a microfabricated flow cytometer for the discrimination of micron-sized polymer beads [92]. Fluorescently labeled proteins were immobilized on the beads, which can be used to analyze the immune response. Negative dielectrophoretic force was used to focus the polymer beads by the focusing electrodes. An electrical impedance flow cytometer for the high-speed analysis of particles was developed [93]. The impedance signal of polystyrene beads could be obtained in as little as 1 ms. Compared with microchannels fabricated by soft lithography, Kummrow et al. used ultraprecision milling technology to design 3D microchannels with horizontal and vertical focusing capabilities [94]. Fiber optics, mirrors, and electrodes were integrated into a flow cytometer for blood cells. Spencer et al. conducted a study on how the position of particles in a microchannel affects impedance measurements [95]. Impedance is related to the position of the particle in the vertical direction. A flow cytometer was designed by Barat et al. in order to measure both the optical and electrical properties of particles [96]. Daniel Spencer integrated optical fibers and waveguides into an impedance flow cytometer to measure electrical impedance (electrical volume and opacity), fluorescence, and large-angle side scatter without the need for particle focusing [97]. Haandbæk et al. published an article on microfabricated flow cytometry at high frequencies [98]. The experimental results indicated the ability at high frequencies to distinguish not only wild-type yeast and mutant strains but also opacity values at frequencies above 50 MHz.

**Figure 7:**
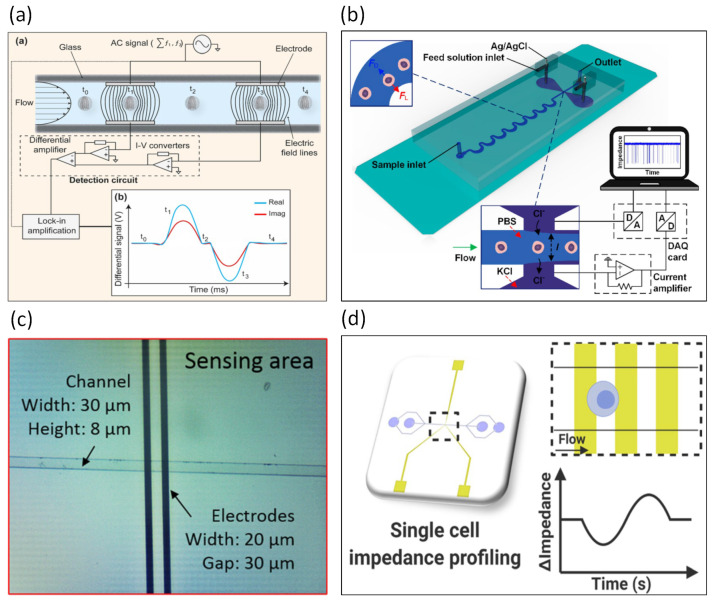
Dynamic cell analyzed by microfluidic impedance cytometry. (**a**) A model of the impedance flow cytometer was established to distinguish Cryptosporidium parvum, Cryptosporidium muris, and Giardia lamblia in minutes. Reproduced with permission from [104]. Copyright Scientific Reports 2017. (**b**) A microfluidic impedance cytometer integrating inertial focusing and liquid electrodes was developed. Reproduced with permission from [105]. Copyright Analytical Chemistry 2017. (**c**) An impedance flow cytometer was developed to detect spheroid green algae cells (Picochlorum SE3) at different salt concentrations. Reproduced with permission from [108]. Copyright Scientific Reports 2020. (**d**) A microfluidic impedance cytometer was used for the analysis of antigen-specific T lymphocytes. Reproduced with permission from [110]. Copyright Sensors and Actuators B: Chemical 2021.

David et al. used impedance flow cytometry to measure the viability and membrane potential of *Bacillus megaterium* cells [99]. A microfluidic impedance cytometer was designed for platelet analysis by Evander et al. [100], where the focusing electrodes allow for secondary focusing of the sample through the dielectrophoretic force. Lin et al. used a microfluidic impedance cytometer to detect quantified protein biomarkers [101]. For whole blood analysis, Simon et al. developed a microfabricated AC impedance cytometer with multi-frequency AC impedance and side scatter analysis capabilities [102]. Xie et al. proposed the concept of using a microfabricated impedance cytometer to detect electronic biomarkers [103]. By changing the dielectric properties of the particle, the authors designed a nanoelectronic barcode particle as an electronic biomarker. McGrath et al. used a microfluidic impedance flow cytometer to distinguish the oocysts of protozoan parasites, as shown in Figure 7a [104]. This chip can distinguish between *Cryptosporidium parvum*, *Cryptosporidium muris*, and *Giardia lamblia* within minutes. A microfluidic impedance cytometer integrating inertial focusing and liquid electrodes was developed for the high-throughput measurement of human breast tumor cells and leukocytes, as shown in Figure 7b [105]. The purpose of inertial focusing is to reduce cell adhesion and ensure that single cells pass through the sensing area. An interesting study involved the use of two microneedles, placed on either side of a microchannel to sense impedance values [106]. For clinical analysis and judgment, Sun et al. used multi-frequency impedance spectroscopy and machine learning to rapidly distinguish the survival of cancer cells under the action of anti-matriptase-conjugated drugs [107]. Sui et al. developed an impedance flow cytometer to detect spheroid green algae cells (Picochlorum SE3) at different salt concentrations, as shown in Figure 7c [108]. Mahesh et al. published a study on the observed “double-peak” characteristics of individual cells with high sensitivity to changes in cell membrane capacitance [109]. This phenomenon has limitations: it operates at the lower frequencies (400–800 kHz) of the beta-dispersion mechanism, and the microelectrodes must be coplanar and paired. The authors pointed out that changes in cell size and membrane capacitance can be resolved using a single frequency. A microfluidic impedance cytometer was used for the analysis of antigen-specific T lymphocytes, as shown in Figure 7d [110]. The experimental results demonstrated that differences in impedance can be observed among dead, healthy, and activated lymphocytes. Caselli et al. used artificial intelligence methods to decipher signals from microfluidic impedance cytometers [111]. The authors demonstrated two advances: (i) the use of a neural network to determine the dielectric properties of single cells in raw impedance data streams and (ii) resolving the impedance signatures of coincident cells. The results demonstrated that the neural network could increase the signal processing capability of the microfluidic impedance cytometer.

### 5.5. Detection of Viruses

The key to the use of impedance biosensors for virus sensing is the immobilization of antibodies, where the antibody must be immobilized on the surface of the electrode first. However, the impedance signal will be altered due to the binding of both antibodies and antigens.

**Figure 8:**
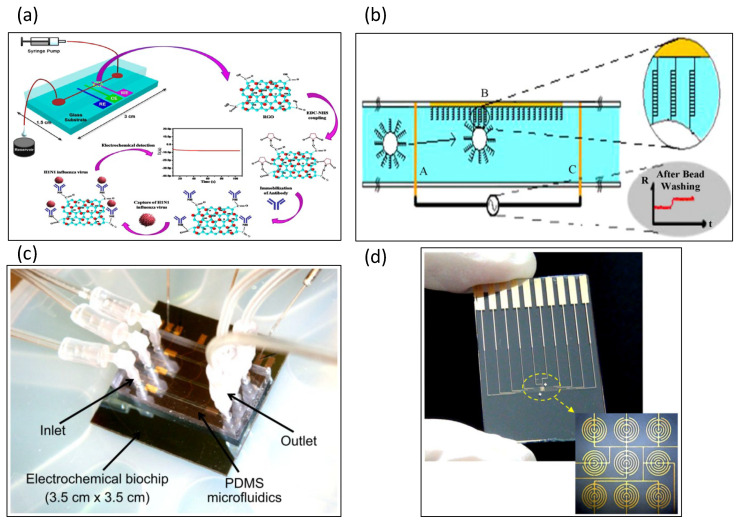
Glass-based impedance biosensors for detecting other organisms and chemicals. (**a**) An electrical impedance sensing chip was developed to detect influenza H1N1 virus. Reproduced with permission from [115]. Copyright Scientific Reports 2017. (**b**) Impedance sensors were developed for DNA hybridization. Reproduced with permission from [117]. Copyright Sensors and Actuators B: Chemical 2011. (**c**) Single-stranded DNA probes were functionalized onto electrodes. Complementary DNA hybridization was then induced using electrochemical impedance spectroscopy. Reproduced with permission from [118]. Copyright Biosensors and Bioelectronics 2012. (**d**) Galectin-1 protein is a biomarker of bladder cancer. An impedance immunosensor was developed to detect Galectin-1 protein in urine. The dielectrophoretic force was used to capture Galectin-1 antibody to improve the sensitivity of the sensor. Reproduced with permission from [119]. Copyright Biosensors and Bioelectronics 2016.

Pathogenic avian influenza viruses have been detected by impedance sensors [112]. The polyclonal antibody was immobilized on the surface of a gold microelectrode by protein A. The antibody–antigen binding reaction in the same research could be amplified by red blood cells, and the proposed impedance immunosensor could be completed in as little as 2 h. A portable impedance biosensor for avian influenza virus H5N2 was developed by Wang et al. [113]. Magnetic nanobeads and interdigitated array microelectrodes were integrated on a microfluidic chip, where the magnetic nanobeads captured avian influenza virus subtype-specific antibodies. The entire detection time was less than 1 h, which was greatly reduced compared with traditional avian influenza virus detection. The experimental results indicate that the sensitivity of the impedance biosensor is comparable to that of real-time reverse transcriptase PCR. Jacob Lum designed an impedance biosensor, mainly for avian influenza virus subtype H5N1 [55]. For this purpose, immunomagnetic nanoparticles and interdigitated microelectrodes were designed on a microfluidic chip. The polyclonal antibody was immobilized on the surface of the microelectrode, which binds to avian influenza virus H5N1 to generate an impedance signal. Chicken red blood cells were used as biomarkers attached to interdigital microelectrodes. The results showed that the impedance signal can be increased by more than 100%. For the detection of human immunodeficiency virus, Shafiee et al. designed a microfluidic sensing chip to measure the impedance spectrum of virus nanolysates [114]. Two gold electrodes were designed on both sides of the microchannel cavity. The lysis of the virus results in the release of the ions and charged molecules of the virus into a non-ionic solution. That research demonstrated that impedance spectroscopy provides a convenient and rapid tool for the detection of multiple pathogens. Singh et al. developed an electrical impedance sensing chip to detect influenza H1N1 virus, as shown in Figure 8a [115,116], for which three microelectrodes were fabricated on a glass substrate. The authors used reduced graphene oxide and monoclonal antibodies as electrode modifications.

### 5.6. Detection of Other Analytes and Chemicals

Berdat et al. used an impedance sensor based on an interdigitated microelectrode array to sense DNA [116], for which 5 µm wide microelectrodes were fabricated using a lift-off process method. The complementary probe was first immobilized on the electrode and hybridized to the target ssDNA. Finally, impedance sensors were used to detect the pathogen *Salmonella choleraesuis* in dairy products. Javanmard et al. developed a set of impedance sensors for DNA hybridization, as shown in Figure 8b [117]. Oligonucleotide probes were immobilized on the surface of the microchannel, and target DNA strands were immobilized on the surface of polystyrene beads. Contact between the probe and the target DNA strand results in the hybridization of the DNA, leading to the capture of the polystyrene beads on the surface of the microchannel. An impedance chip for sensing DNA hybridization was developed by Hadar et al., as shown in Figure 8c [118]. Single-stranded DNA probes were functionalized onto electrodes, and complementary DNA hybridization was then induced using electrochemical impedance spectroscopy. DC-biased AC electro-osmotic vortex was utilized to design a label-free electrochemical impedance spectroscopy (EIS)-based DNA biosensing chip [119]. Based on the electro-osmotic vortex, 20-base target DNA fragments were hybridized to achieve 90% within 141 s. The ultrasensitive detection limit was 0.5 aM. Another study indicated that the electric field was manipulated by alternating current (AC) electrokinetics to improve hybridization efficiency and reduce hybridization time [120]. Thus, the chip was realized for faster and more efficient detection.

Galectin-1 protein is a biomarker of bladder cancer. An impedance immunosensor for detecting bladder cancer in urine was developed, as shown in Figure 8d [121]. Before measuring the impedance signal, the authors used dielectrophoretic force to capture nanoprobes (Gal-1 antibody) on the surface of the microelectrode, in order to improve the sensitivity of the sensor. Alsabbagh et al. designed a microfluidic impedance biosensor for the detection of myocardial infarction proteins by electrochemical impedance spectroscopy [122]; in particular, Troponin I, which is a biomarker for the diagnosis of myocardial infarction, was targeted. Self-assembled thiolated oligonucleotides tested on gold electrodes were found to perform better, as they improved the performance of the impedance signal. Fluorescence analysis and electrochemical impedance spectroscopy were integrated to measure aggregated C-reactive proteins [123]. The circular array of electrodes was designed to create electrokinetic flow for C-reactive protein aggregation. Interdigitated microelectrode arrays were modified by the self-assembled monolayers of mercaptocaproic acid for detecting the arthritis anti-CCP-ab biomarker [124]. The experimental results showed that the sensor response increased linearly with the stepwise increase of the biomarker concentration. A polyaniline (PANI)/MoS2-modified screen-printed electrode was detected for anti-cyclic citrullinated peptide [125]. Among them, the polymerized PANI-Au nanomatrix was utilized to entrap the aCCP antibodies for amplification of the higher signal. A peptide-based electrochemical sensor was used to detect autoantibodies for the diagnosis of rheumatoid arthritis [126]. The developed peptides were modified on the gold surface of the working electrode by a self-assembled monolayer method. Subsequently, the sensor was clinically demonstrated to have better sensitivity using 10 clinically validated samples from rheumatoid arthritis patients and 5 healthy control samples.

Chiriaco et al. designed a microfluidic electrochemical impedance biosensor to detect cholera toxin [127] with sensitivity less than 10 pM. Liu et al. developed a biosensor for the rapid screening of toxic substances in drinking water [128]. Cells are damaged due to toxins in water, resulting in decreased impedance and an increase in resonance frequency. Impedance and mass sensing measurements can help to improve sensor accuracy. An impedance biosensor was used to detect the cytotoxicity of tamoxifen in cervical cancer cells [129]. The experimental results showed that the dose of tamoxifen resulted in a significant reduction in the number of HeLa cells. The same article demonstrated that impedance biosensors can be used for the evaluation of novel drugs and cytotoxicity.

A microfluidic impedance sensor was designed for pesticide detection in vegetables [130]. Anti-chlorpyrifos monoclonal antibody was immobilized on an interdigitated electrode array. The capture of chlorpyrifos produced a change in impedance. Zeng et al. integrated magnetic focusing into impedance microsensors for oil monitoring [131]. The highly focused magnetic field was derived from two electromagnetic coils and eight silicon steel tips, where the silicon steel tips greatly improved the sensitivity of the sensor.

### 5.7. Conclusions of the Glass Chips

Microfluidic impedance sensor glass chips have two great development directions: electrochemical impedance spectroscopy and electrical impedance flow cytometry. The difference between the two lies in the state of the measured object. For example, in an electrical impedance flow cytometer, as shown in Figure 6, the cells are stationary. The cells may be attached to the bottom of a microgroove or a microchamber, where the culture medium is quiescent. On the other hand, cells may be trapped by the microstructures and focused by the dielectrophoretic force, thus, fixing them in one position. In an electrical impedance flow cytometer, the cells are in a dynamic state, as shown in Figure 7. For the impedance sensing of other organisms, the main technology relies on the modification of electrodes and the combination of antigens and antibodies.

## 6. Paper-Based Impedance Biosensors

**Figure 9:**
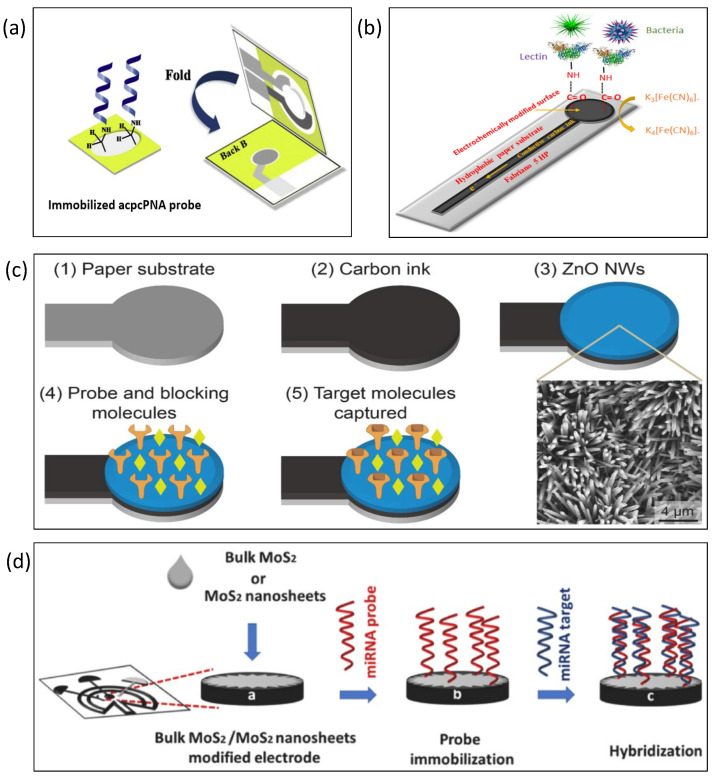
Paper-based impedance biosensors. (**a**) A paper-based electrochemical impedance DNA sensor was developed for tuberculosis detection. Reproduced with permission from [133]. Copyright Analytica Chimica Acta 2018. (**b**) Paper-based electrodes for impedance sensors were used to detect the bacteria in water. Reproduced with permission from [134]. Copyright Sensors and Actuators B: Chemical 2018. (**c**) A paper-based electrochemical impedance spectroscopy was designed to detect coronavirus (COVID-19). Reproduced with permission from [139]. Copyright Biosensors and Bioelectronics 2021. (**d**) Paper-based impedance sensor was used to sense miRNA-155 and miRNA-21 for early diagnosis of lung cancer. Reproduced with permission from [143]. Copyright Talanta 2022.

Lei et al. combined the electrical impedance measurement technique with the hydrophilic properties of a paper base into a system for recording and analysis [132]. Through impedance analysis, a trend proportional to cell proliferation was observed. A paper-based electrochemical impedance DNA sensor for tuberculosis detection was developed by Teengam et al., as shown in Figure 9a [133]. Carbon graphene inks were printed as working and counter electrodes, while reference electrodes and conductive pads were screen-printed with silver/silver chloride ink. Pyrrolidine peptide nucleic acid (acpcPNA) was immobilized on cellulose paper, and changes in impedance were induced in the presence of *Mycobacterium tuberculosis*. Rengaraj et al. developed paper-based electrodes for the impedance detection of bacteria in water, as shown in Figure 9b [134]. The paper-based electrodes were made of carbon on hydrophobic paper formed by screen printing. The cellulose was cross-linked before use in order to enhance the strength of the paper substrate and the electrical properties of the screen-printed electrodes. The same article was the first to combine the hydrophobicity of paper substrates with the electrochemical functionalization of electrodes. A paper-based microfluidic impedance chip was developed to sense alpha-fetoprotein in human serum using peptide-modified plastic paper [135]. The sensor included two layers, where the upper layer was cellulose chromatography paper, and the lower layer was plastic paper. Among them, the sensing electrodes were printed with Ag-20 wt % graphene. The limit value of alpha-fetoprotein in PBS detected by the sensor was 1 ng/mL. Lei et al. used hydrogel to encapsulate cells and then cultured them on top of paper substrates [136]. The resulting analysis could more accurately distinguish the impedance differences between two cells and under the action of a drug. Some studies have used electrochemical impedance spectroscopy to detect miRNA-34a, which is a biomarker of cancer and Alzheimer’s disease [137]. PAMAM dendrimers were modified on the surface of screen-printed electrodes. The results indicated that the difference between miRNA-34a and miRNA-15 or miRNA-660 could be distinguished through electrochemical impedance spectroscopy. Cardiac troponin I is a biomarker for the early diagnosis of acute myocardial infarction. A paper-based impedance immunosensor was developed to detect cardiac troponin I [138], for which multi-walled carbon nanotubes were immobilized on carbon ink electrodes. Then, cardiac troponin I antibody was immobilized on multi-walled carbon nanotubes. Finally, cardiac troponin I was captured by the antibody, affecting the impedance signal. Li et al. designed paper-based electrochemical impedance spectroscopy to detect coronavirus (COVID-19), as shown in Figure 9c [139]. The carbon ink was first printed on paper, and a layer of zinc oxide nanowires was grown on the carbon ink. Then, probes and blocking molecules were immobilized on its surface. Finally, the target molecules were captured for impedance sensing. The results showed that the enhanced ZnO nanowires can improve impedance sensitivity. Electrochemical impedance spectroscopy was used to analyze artificial sweat using hand-painted electrodes [140]. Electrodes were drawn on the opposite side of the paper in order to reduce the double-layer capacitance. The silver electrode pattern paper chip had stronger impedance stability than the graphite electrode paper chip. Research and development of paper-based electrochemical impedance spectroscopy was conducted for the detection of microRNA 155 [141], for which gold nanoparticles changed the properties of paper-based electrodes. S. Karuppiah developed a paper-based impedance biosensor for monitoring bacteria in water [142]. The electrodes of the biosensor were screen-printed with graphene (G) and then surface-modified with graphene oxide (GO). The lectin concanavalin A was then immobilized on the modified electrode described above. A paper-based impedance sensor was used to sense miRNA-155 and miRNA-21 for the early diagnosis of lung cancer, as shown in Figure 9d [143]. The authors used MoS2 crystals and MoS2 nanosheets to modify the paper-based electrodes. Paper-based electrochemical impedance spectroscopy was used to detect foodborne pathogens (Listeria) [144]. Tungsten disulfide nanostructures were used as paper-based electrodes for impedance sensors.

Paper-based chips have great advantages due to their low material cost. Microelectrodes with small line widths on paper-based chips and antibodies combined onto the microelectrodes will be key technologies for the development of paper-based chips.

## 7. Stretchable Biosensors

**Figure 10:**
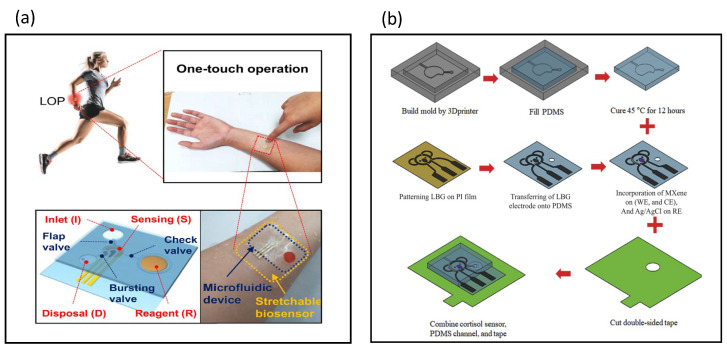
Stretchable impedance biosensors. (**a**) All-polymer electrochemical microfluidic biosensors were used for sensing the biomarker cortisol in sweat. The pullable body impedance biosensor was designed at the bottom layer and attached to the skin. Reproduced with permission from [147]. Copyright Biosensors and Bioelectronics 2020. (**b**) A wearable microfluidic impedance immunosensor was designed for sweat cortisol detection. Microfluidic channels and chambers were integrated into the wearable patch. Reproduced with permission from [149]. Copyright Sensors and Actuators B: Chemical 2021.

Furniturewalla et al. designed a microfluidic impedance cytometer on a flexible circuit board in the form of a portable wristband [145]. Lock-in amplification, a microfluidic biosensor, a microcontroller, and a Bluetooth module are integrated into the wristband. Flexible and stretchable biosensors for skin physiological parameter monitoring have been developed [146], where screen printing was used to fabricate sensing electrodes in flexible and stretchable conductive materials originally intended for epidermal tattooing. A retractable body biosensor for sensing the biomarker cortisol in sweat was published, as shown in Figure 10a [147]. A pullable body impedance biosensor was designed at the bottom layer and attached to the skin, following which microfluidic microvalves and microchannels were applied to this wearable patch. A stretchable microfluidic immunobiosensor patch was used for sensing neuropeptide Y in human sweat [148]. Conductive microfibers that can be stretched help to improve the sensitivity of the biosensor patches. Sensors attached to the skin can detect biomarker concentrations in human sweat at levels as low as fm. A wearable microfluidic impedance immunosensor for sweat cortisol detection was designed, as shown in Figure 10b [149], where microfluidic channels and chambers were integrated into the wearable patch, and Ti3C2Tx MXene nanosheets were incorporated in the porous structure of graphene. The wearable microfluidic impedance immunosensor could detect cortisol down to 88 pM.

Stretchable chips for microfluidic impedance sensors comprise a novel research direction. At present, impedance sensing signals are mostly measured on the skin. The stretchability and bendability of such materials are key features, especially the flexibility of the electrode materials. In this line, conductive hydrogels may be a helpful technology.

## 8. Conclusions

Impedance biosensors integrated with microfluidic technology are powerful tools for understanding electrical information in microscopic and sub-microscopic organisms. The integrated sensors have the key characteristics of improved sensitivity, reduced reagent consumption, short analysis time, reduced instrument size, and simple operation. In this paper, the developed microfluidic impedance sensors were classified into six categories: silicon chip-, PCB chip-, polymer chip-, glass chip-, paper chip-, and stretchable chip-based. No matter which type of chip is to be developed, the microfabrication of microelectrodes and the bonding of chemicals are key technologies.

Silicon-based chips are made by the MEMS process developed by Taiwan Semiconductor Manufacturing Co., Ltd., which can become the commercialization direction of electrical impedance biosensors. The development of PCB chips was limited by the line width of the electrodes, which makes it impossible to improve sensitivity. Therefore, PCB chips are more suitable for production or commercial designs with a fast sensing speed and low sensitivity requirements. Polymer materials are suitable for mass production. Therefore, polymer chips can be one of the options for commercialization. Microfluidic impedance sensor glass chips have two great development directions: electrochemical impedance spectroscopy and electrical impedance flow cytometry. The difference between the two lies in the state of the measured object. In an electrical impedance flow cytometer, the cells are stationary. In an electrical impedance flow cytometer, the cells are in a dynamic state. For the impedance sensing of other organisms, the main technology relies on the modification of electrodes and the combination of antigens and antibodies.

Since the development of microfluidic impedance sensors, the development of the principle has mostly stagnated since 2014, especially electrical impedance flow cytometers. Therefore, with regard to the development of sensing applications, specific antigen–antibody binding may not easily to become a break-point in relevant research. Instead, paper-based chips and stretchable chips are expected to become the focus of future development related to microfluidic impedance sensors. As the COVID-19 virus has spread globally in recent years, this variable virus needs to be sensed by electrical impedance biosensors.

## Data Availability

Not applicable.
